# Effect of Microstructural Change under Pressure during Isostatic Pressing on Mechanical and Electrical Properties of Isotropic Carbon Blocks

**DOI:** 10.3390/ma17020387

**Published:** 2024-01-12

**Authors:** Tae-Sub Byun, Sang-Hye Lee, Suk-Hwan Kim, Jae-Seung Roh

**Affiliations:** School of Materials Science and Engineering, Kumoh National Institute of Technology, Daehak-ro 61, Gumi 39177, Republic of Korea; qxt1026@kumoh.ac.kr (T.-S.B.); shlee3106@kumoh.ac.kr (S.-H.L.); sukhwan@kumoh.ac.kr (S.-H.K.)

**Keywords:** cold isostatic pressing, carbon block, pressure, isotropic ratio, mechanical property

## Abstract

In this study, carbon blocks were fabricated using isotropic coke and coal tar pitch as raw materials, with a variation in pressure during cold isostatic pressing (CIP). The CIP pressure was set to 50, 100, 150, and 200 MPa, and the effect of the CIP pressure on the mechanical and electrical properties of the resulting carbon blocks was analyzed. Microstructural observations confirmed that, after the kneading, the surface of isotropic coke was covered with the pitch components. Subsequently, after the CIP, granules, which were larger than isotropic coke and the kneaded particles, were observed. The formation of these granules was attributed to the coalescence of kneaded particles under the applied pressing pressure. This granule formation was accompanied by the development of pores, some remaining within the granules, while others were extruded, thereby existing externally. The increase in the applied pressing pressure facilitated the formation of granules, and this microstructural development contributed to enhanced mechanical and electrical properties. At a pressing pressure of 100 MPa, the maximum flexural strength was achieved at 33.3 MPa, and the minimum electrical resistivity was reached at 60.1 μΩm. The higher the pressing pressure, the larger the size of the granules. Pores around the granules tended to connect and grow larger, forming crack-like structures. This microstructural change led to degraded mechanical and electrical properties. The isotropic ratio of the carbon blocks obtained in this study was estimated based on the coefficient of thermal expansion (CTE). The results confirmed that all carbon blocks obtained proved to be isotropic. In this study, a specimen type named CIP-100 exhibited the best performance in every aspect as an isotropic carbon block.

## 1. Introduction

Graphite blocks are thermally and chemically resistant while also demonstrating high electrical conductivity. They are generally categorized into those with isotropy and others with anisotropy [1,2,3]. Isotropic graphite blocks exhibit uniform and high density, as well as high strength at high temperatures, and thus are widely used as raw materials in various applications, including mechanical seals, semiconductor crucibles, discharge electrodes, and nuclear reactors [4,5,6,7]. The properties of these isotropic graphite blocks are consistent regardless of the direction, with an isotropic ratio ranging from 0.90 to 1.10 [1,8]. In general, the closer to 1.00 the isotropic ratio is, the smaller the dimensional differences across directions become. This characteristic ensures that isotropic graphite blocks have a longer service life when used as high-temperature structural components [9].

Isotropic graphite blocks are typically fabricated using isotropic coke as a filling material and pitch as a binder. During the fabrication process, all these raw materials undergo a series of sequential processes, including mixing, kneading, milling, forming, carbonization, impregnation, re-carbonization, and finally, graphitization. Carbon blocks are intermediate products that have undergone processes leading up to carbonization. They are also used as final products intended for use at around 1000 °C. The forming process throughout the entire procedure is conducted in either unidirectional pressing mode or cold isostatic pressing (CIP) mode. The choice of mode determines the directional strength and electrical conductivity of the resulting carbon blocks and graphite blocks [1,10,11,12,13,14].

The CIP process involves subjecting the material to pressure from all directions, ultimately leading to uniform and dense formation. This mode is suitable for forming processes intended for high-density, high-strength pressed bodies. In general, blocks fabricated in CIP mode are commonly referred to as isotropic blocks [15,16,17,18,19]. The process parameters for CIP include mold thickness, pressing time, and pressure [19,20,21]. Typically, the maximum pressure applied during the CIP process for fabricating carbon blocks is 200 MPa [1,13].

Most of the studies on CIP have been reported in the field of metals and ceramics [22,23,24], among which there are some reports on density and porosity changes with CIP pressure in the field of ceramics [20,21]. Studies using CIP to manufacture isotropic carbon blocks and graphite blocks have been reported in the past but are extremely uncommon [25,26]. He et al. prepared isotropic graphite blocks by CIP and reported property changes by varying the ratio of coke to natural graphite [25]. Yeo et al. prepared isotropic graphite blocks by unidirectional pressing and CIP and reported differences in properties depending on the molding method [26]. Previously reported studies have only used the CIP method to produce isotropic graphite blocks but have not optimized the process conditions for CIP. Especially, little research has focused on the fabrication of isotropic carbon blocks and graphite blocks with variations in CIP pressure. Moreover, there has been little research interest directed toward the effect of CIP pressure on their mechanical and electrical properties, along with the supporting microstructural analysis for such interpretations.

In this study, carbon blocks were fabricated in CIP mode using isotropic coke and coal tar pitch, and the effect of CIP pressure on their properties was examined. The porosity, mechanical properties, and electrical characteristics of the resulting carbon blocks were measured. The obtained results were then integrated with the observed microstructural evolution for further analysis and interpretation. Additionally, the CTE was measured to determine the degree of isotropy in the carbon blocks.

## 2. Experimental Procedure

### 2.1. Raw Materials and Preparation

In this study, commercial isostatic coke (POSCO MC MATERIALS, Jeonnam, Republic of Korea) was used as the filler material, with an average particle size of 6.73 µm. The binder was a commercial coal tar pitch (Rain Carbon Inc, Stamford, CT, USA). Coal-based pitch has a softening point of 110 °C, quinoline insoluble ranging from 4 to 8%, toluene insoluble ranging from 22 to 28%, ash 0.3%, and a coking value of 54%. The filling material and binder were mixed at a weight ratio of 75:25, and the mixture was then kneaded at 170 °C for 30 min. Subsequently, the kneaded mixture was pulverized. A total of 255 g of the obtained powder was fed into urethane molds and then subjected to CIP for 30 min with pressures of 50, 100, 150, and 200 MPa. Three isotropic carbon blocks were prepared for each CIP pressure condition, and it was found that the deviation of density after surface treatment was small. Among them, a carbon block with a medium value was selected to check the uniformity within it.

**Figure 1:**
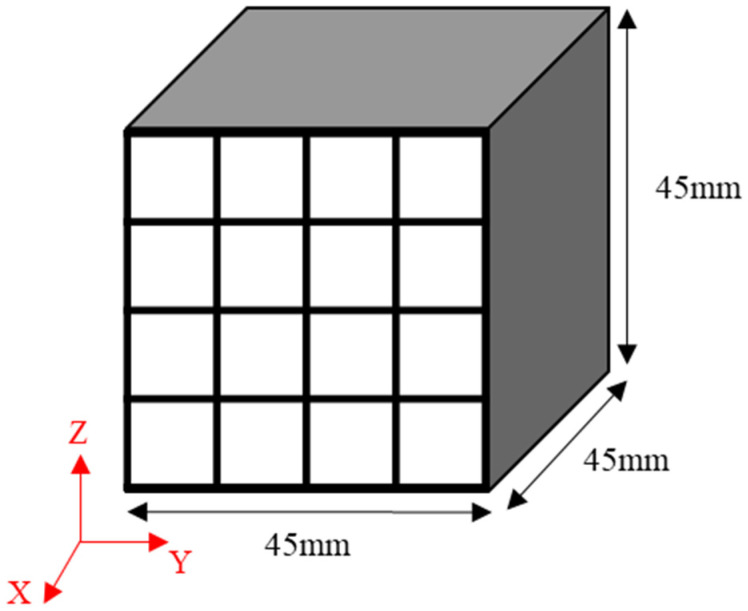
Schematic illustration of the sampling strategy applied: 9 × 9 × 45 mm^3^ bodies sampled from a 45 × 45 × 45 mm^3^ isotropic carbon block.

The obtained green bodies subsequently underwent carbonization in a tube furnace in which an inert atmosphere was achieved using nitrogen gas. They were heated to 1000 °C and then maintained at that temperature for one hour [2,27]. Each isotropic carbon block was cut into 16 blocks with dimensions of 9 × 9 × 45 mm^3^ to measure the bulk density, mechanical properties, and electrical characteristics at different locations and determine their deviation, as shown in Figure 1. The mechanical properties of each of the 16 blocks were separately measured, averaged, and then used to determine the deviation.

**Figure 2:**
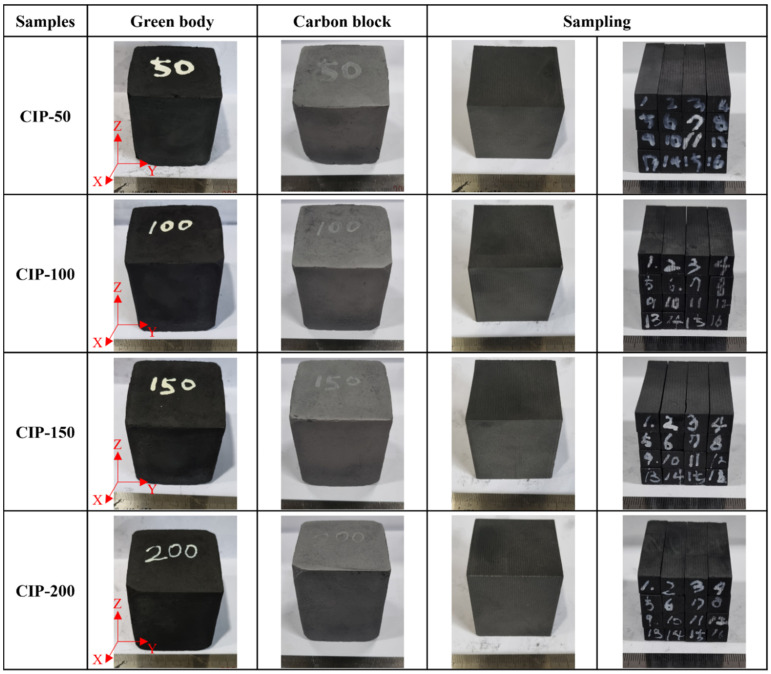
Images of the isotropic carbon blocks fabricated under different CIP pressures.

**Figure 3:**
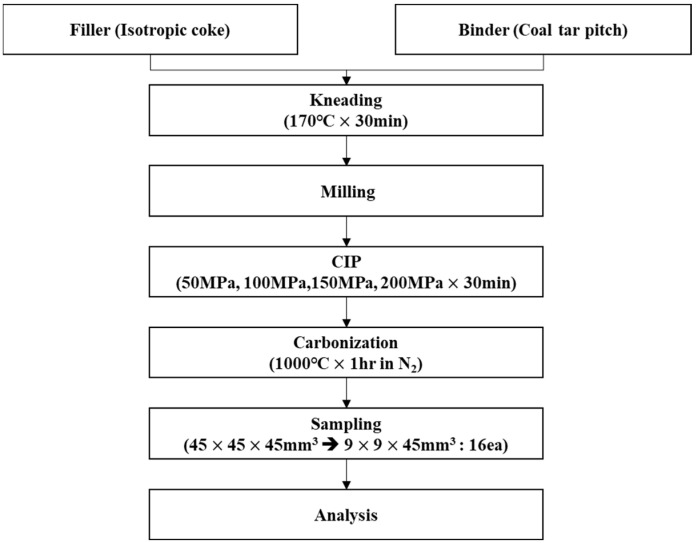
Schematic representation of the experimental procedure.

The samples were named CIP-50, CIP-100, CIP-150, and CIP-200 with respect to CIP pressure. Figure 2 shows images of the isotropic carbon blocks fabricated under different CIP pressures, and the overall experimental procedure is shown in Figure 3.

### 2.2. Observation of Isotropic Coke and Kneaded Particles

Scanning Electron Microscopy (SEM, MAIA 3 LM, TESCAN, Brno, Czech Republic) was employed to analyze the overall particle shape and size of isotropic coke as a raw material, along with the kneaded particles.

### 2.3. Bulk Density and Porosity Measurement

The bulk density and porosity of the cut isotropic carbon blocks were measured by the standards provided in ISO 18754:2020 [28]. The cut surface of each isotropic carbon block was polished with #1500 sandpaper, subjected to ultrasonic cleaning, and then dried in an oven at 60 °C for 48 h. The weight of the dried specimens was then measured. Afterward, the dried block specimens were boiled in distilled water for three hours. They were then cooled at room temperature before measuring both the underwater weight and saturated weight. Bulk density and porosity were measured using the equations below [2].

Bulk density (g/cm^3^) = Dry weight/(Saturated weight − Underwater weight) (1)

Porosity (%) = {(Saturated weight − Dry weight)/(Saturated weight − Underwater weight)} × 100(2)

The deviation of density was calculated using the equation below, referencing the maximum and minimum values obtained from the bulk density measurements of the cut isotropic carbon blocks with respect to CIP pressures.

Density deviation (g/cm^3^) = Maximum bulk density − Minimum bulk density (3)

### 2.4. Microstructure Observation

The microstructure of the isotropic carbon blocks was analyzed using SEM and optical microscopy (OM, Nikon ECLIPSE, Tokyo, Japan). Out of the 16 cut carbon blocks, those that were utilized to estimate the average bulk density were used for microstructural analysis. To this end, the cross-sectional surface perpendicular to the x-axis of each of these blocks was finely polished (refer to Figure 1). OM was employed to identify two points that represented the overall characteristics of the microstructure, and these two points were photographed at a magnification of 100×. Subsequently, SEM was utilized to obtain enlarged images of these points.

### 2.5. Flexural Strength Measurement

Flexural strength tests were performed on the cut isotropic carbon blocks using a universal testing machine by the three-point bending test method provided in ASTM D 7972 [29]. The bending speed was set to 0.5 mm/min, and the flexural strength was calculated using the equation below [2].

Sb = 3WI/2bt^2^ (4)

Sb: flexural strength (N/cm^2^), I: distance between the two points (cm), W: maximum load, b: specimen width (cm), and t: specimen thickness (cm).

### 2.6. Shore Hardness Measurement

The Shore hardness of the isotropic carbon blocks was measured using an indicator type (D type) according to the specifications provided in ASTM C 886 [30]. Each specimen underwent a total of six measurements, with the average value serving as the Shore hardness measurement.

### 2.7. Electrical Resistivity Measurement

The electrical resistivity of the isotropic carbon blocks was measured using the voltage drop method provided in ASTM C 611 [31]. The isotropic carbon block was placed against a copper plate at each end, the specimen was fixed to prevent it from moving, and the power supply (GW INSTEK, GPE-3323, New Taipei City, Taiwan) was used to apply currents of 0.5, 1.0, 1.5, 2.0, 2.5, and 3.0 A. The voltage was measured by contacting a voltage terminal with a distance of 1.6 cm between the terminals to the center of the specimen. The electrical resistivity was calculated, accounting for the measured voltage, the cross-sectional area of the specimen, and the distance between the terminals, according to the equation below.

ρ = eS/il(5)

ρ: electrical resistivity (Ωcm), e: potential drop between the terminals (V), S: cross-sectional area of the specimen (cm^2^), i: current (A), and l: distance between the terminals (cm).

### 2.8. Isotropic Ratio Measurement

In general, carbon blocks with an isotropic ratio range of 0.90–1.10 fall into the category of isotropic carbon blocks [1,8]. The isotropic ratio can be measured by various methods, such as examining the directional characteristics of electrical resistivity and mechanical properties, analyzing crystallinity with XRD analysis, and measuring the CTE values. In this study, the isotropic ratio was assessed based on CTE measurements.

All measurements were performed using a thermomechanical analyzer (TMA, Q400EM, TA Instruments, New Castle, DE, USA). First, of the 16 cut carbon blocks, three that exhibited a bulk density comparable to the average density were selected for CTE measurements. The selected carbon blocks, sampled along the x-axis, y-axis, and z-axis, were heated to 600 °C at a rate of 10 °C/min in an inert atmosphere. During this heating process, CTE measurements were performed in the temperature range of 200–500 °C. Based on the results, the isotropic ratio was calculated using the equation below [1,8].

Isotropic ratio = AG (Against-Grain)/WG (With-Grain)(6)

AG, WG: CTE (μm/m·°C).

## 3. Results and Discussion

### 3.1. Shape and Size of Isotropic Coke and Kneaded Particles

**Figure 4:**
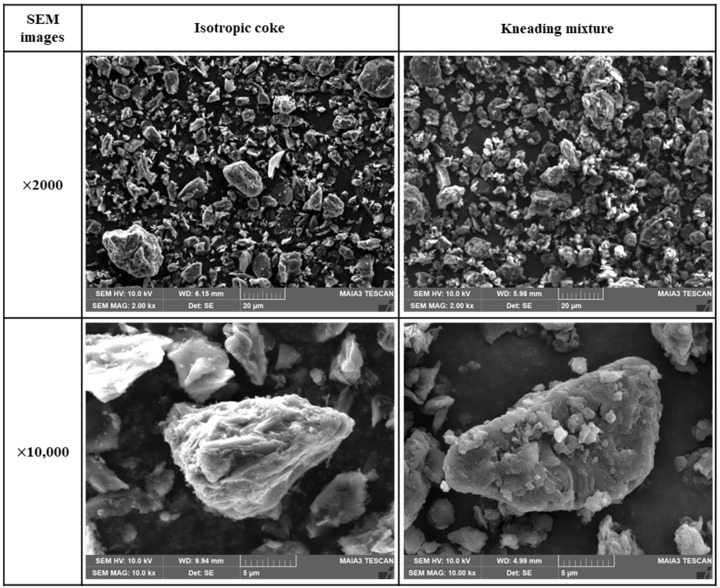
Images of isotropic coke and kneaded particles.

Figure 4 presents SEM images of the isotropic coke and kneaded particles. The isotropic coke particles were found to have sharp edges and rough surfaces. In contrast, the kneaded particles exhibited smooth edges and surfaces compared to the isotropic coke. This observation confirms that the surface of isotropic coke was uniformly covered with pitch in the kneaded mixture specimen. At a magnification of 1000×, it was observed that both isotropic coke and kneaded particles were 20 μm or less in size.

### 3.2. Bulk Density and Porosity

**Figure 5:**
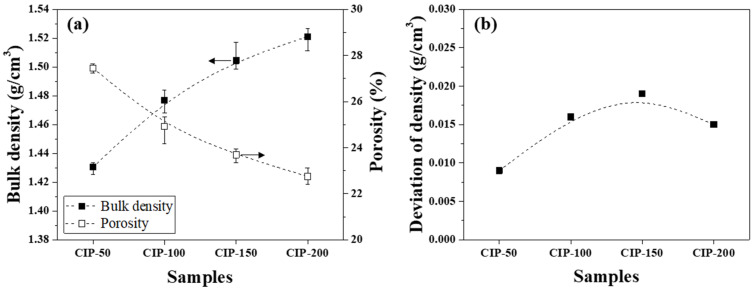
(**a**) Bulk density and porosity and (**b**) deviation of density of isotropic carbon blocks fabricated with respect to CIP pressures.

The bulk density and porosity of the isotropic carbon blocks were measured using 16 sampled bodies, as shown in Figure 5a. Additionally, the deviation between the bulk density measurements was plotted in Figure 5b.

Figure 5a presents changes in the bulk density and porosity of the isotropic carbon blocks with respect to CIP pressure. CIP-50 exhibited a bulk density of 1.431 g/cm^3^ and a porosity of 27.4%. With an increase in CIP pressure, the bulk density increased while the porosity decreased. Indeed, the bulk density and porosity of CIP-200 were 1.521 g/cm^3^ and 22.7%, respectively.

Figure 5b shows the deviation between bulk density measurements with respect to CIP pressure. For all isotropic carbon blocks, the deviation of density was within 0.02 g/cm^3^, regardless of the applied CIP pressure. According to a study previously conducted by the present authors, for graphite blocks fabricated by unidirectional pressing under 150 MPa, the bulk density was 1.395 g/cm^3^, while the deviation of density was 0.027 g/cm^3^ [27]. In this study, CIP-150 exhibited a bulk density of 1.504 g/cm^3^, and the deviation of density was 0.019 g/cm^3^. Compared to when unidirectional pressing was applied, the bulk density of CIP-150 was 7.8% higher, while the deviation of density was 29.6% lower.

### 3.3. Microstructural Analysis

**Figure 6:**
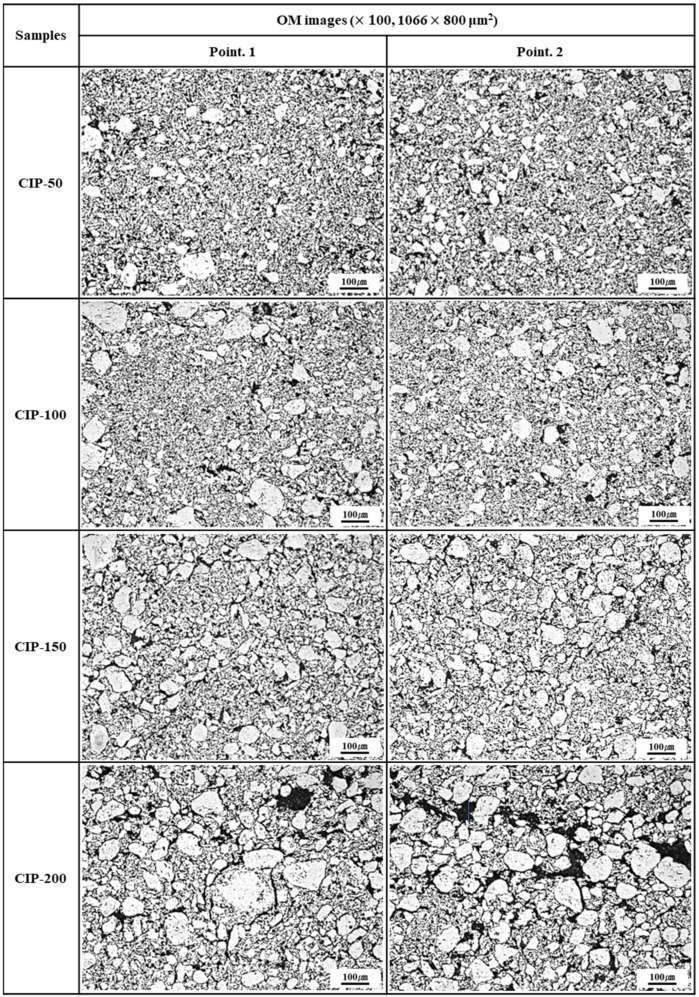
Images of isotropic carbon blocks with respect to CIP pressure (100× by OM).

Figure 6 shows OM images of the isotropic carbon blocks with respect to CIP pressures. The bright particles observed in these OM images were isotropic coke particles or kneaded particles, and they were much larger in size compared to those, as raw materials, observed in Figure 4. These large particles were considered granules, resulting from the coalescence of kneaded particles occurring under CIP pressure.

In CIP-50, the size of the observed granules measured 30–50 μm. Additionally, these large granules were surrounded by smaller ones. In CIP-100 and CIP-150, the average size of granules ranged from 50 to 100 μm. Notably, the number of granules was larger, and their distribution was more uniform in CIP-150 than in CIP-100. Granules tended to grow larger with rising CIP pressure; the particle diameter of CIP-200 measured as large as 100–150 μm.

Furthermore, it was observed that an increase in CIP pressure also led to an increase in the size of porosity, along with variations in its distribution. In CIP-200, pores with sizes ranging between 25 and 30 μm were visible, including some larger than 50 μm.

The observed increase in pore size can be accounted for by the following reasons. With the granules forming and growing, some pores remain within them; however, most of the pores are extruded out of the granules. Consequently, these pores exist around the granules or may coalesce with each other. As the pressing pressure further increases, the size of granules continues to increase, leading to the growth of pores and their connection with each other.

**Figure 7:**
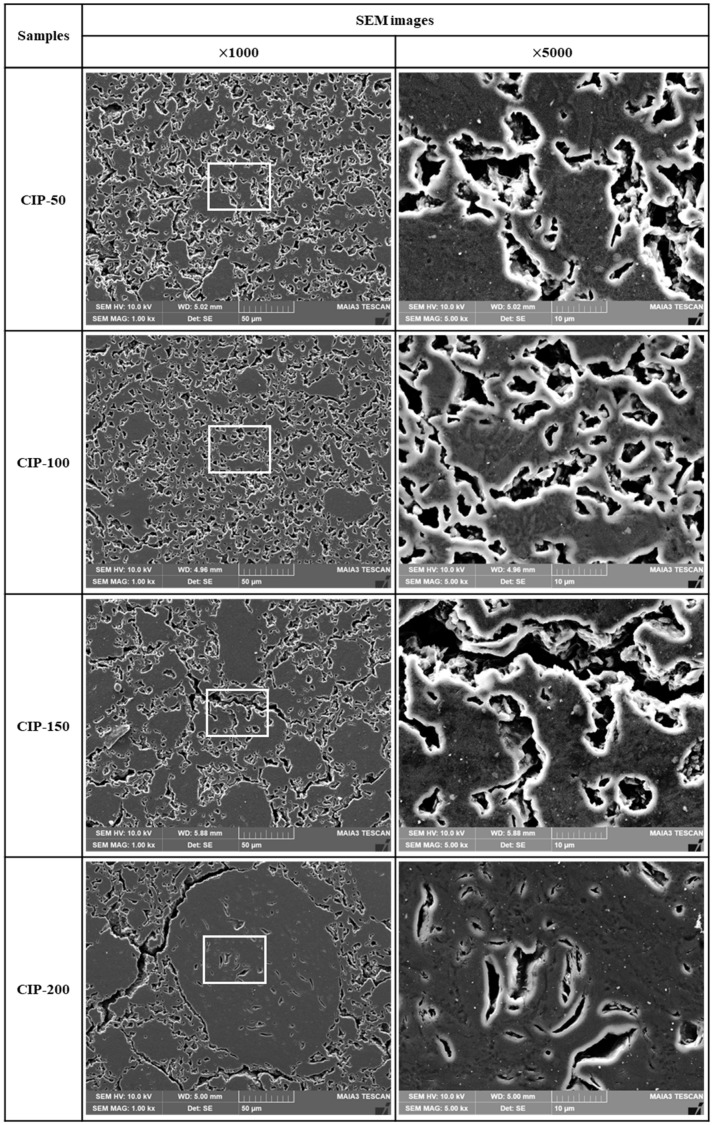
Images of isotropic carbon blocks with respect to CIP pressure (1000× and 5000× by SEM).

Figure 7 shows SEM images of the isotropic carbon blocks fabricated with respect to CIP pressures. As the CIP pressure increased, the growth of granules became more pronounced. In addition to the pores within granules, pores around granules grew larger and connected. In CIP-100, most small pores existed separately without being connected with others, in contrast to specimens with different CIP pressures.

It is expected that the size of granules, along with the size and shape of the associated pores, will affect the mechanical and electrical properties of the resulting carbon blocks. These results will be presented in Section 3.4 and Section 3.5 below.

### 3.4. Flexural Strength and Shore Hardness

**Figure 8:**
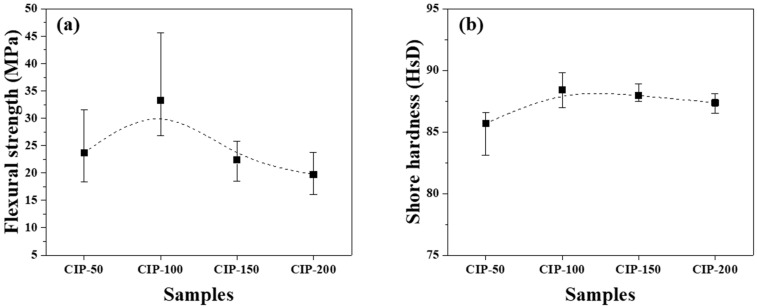
Changes in mechanical properties of isotropic carbon blocks fabricated with respect to CIP pressures for (**a**) flexural strength and (**b**) Shore hardness.

The flexural strength and Shore hardness of the isotropic carbon blocks were measured using the 16 sampled bodies. All measurements were averaged for each condition, and their deviations are plotted in Figure 8.

Figure 8a shows the flexural strength of the isotropic carbon blocks as a function of CIP pressure. CIP-100 achieved the highest flexural strength at 33.3 MPa. With an increase in CIP pressure, the flexural strength decreased, with CIP-200 exhibiting the lowest flexural strength at 19.7 MPa.

Pores are a type of defect in which stress is concentrated. Generally, a decrease in porosity results in an increase in flexural strength [32,33]. Lee et al. previously reported that fractures in graphite blocks propagated along granular boundaries rather than breaking through the granules [34].

Consequently, as the pressing pressure increases, granules grow larger (Figure 6), which is accompanied by the formation of pores around them. Notably, the strength of the resulting carbon block can be affected by the arrangement of these pores. CIP-100 exhibited the highest strength, as shown in Figure 7, likely attributed to the small pores around the granule existing separately without being connected. In CIP-150 and CIP-200, pores were connected to each other, and thus served as crack-like defects, resulting in a degradation in strength.

In Figure 8a, CIP-100 has the highest average value of flexural strength, but the deviation is large. This can be explained by the microstructure. In Figure 6, CIP-100 had fewer and unevenly distributed granules of size between 50 and 100 μm formed. Where the granules were relatively rich, the flexural strength would be lower, and where the granules were relatively poor, the flexural strength would be higher. In Figure 7, CIP-100 showed pores around the granules as crack-like defects, and small pores existed separately in other parts.

Figure 8b shows changes in the Shore hardness of the isotropic carbon blocks with respect to CIP pressure. CIP-100 exhibited the highest hardness at 88.2 HsD, and as the CIP pressure further increased, the hardness decreased. The observed peaking of the Shore hardness, followed by continuous reduction, in CIP-100 clearly aligns with the trend observed in the flexural strength measurements, even though the degree of reduction is smaller in the Shore hardness measurements.

### 3.5. Electrical Resistivity

**Figure 9:**
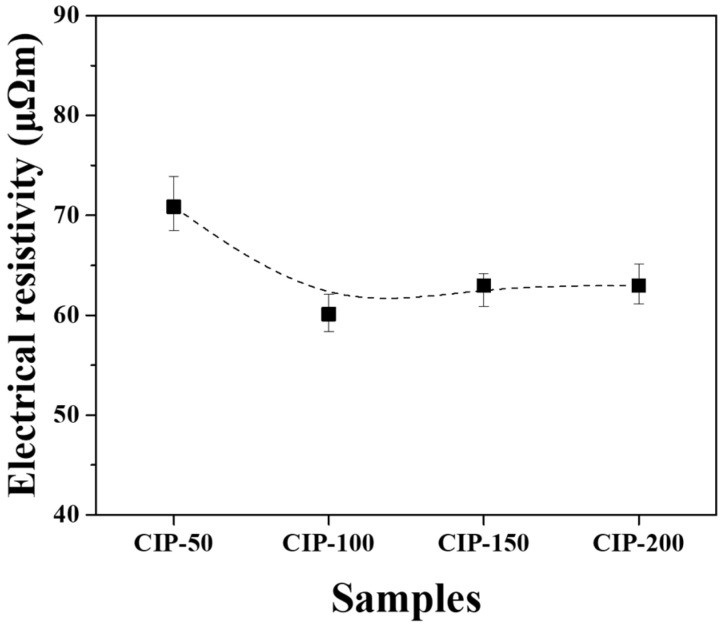
Electrical resistivity of isotropic carbon blocks fabricated with respect to CIP pressures.

The electrical resistivity of the isotropic carbon blocks fabricated with respect to CIP pressures was measured using the 16 sampled bodies. All measurements were averaged for each condition, and their deviations are plotted in Figure 9.

CIP-50 exhibited the highest electrical resistivity at 70.8 μΩm, while CIP-100 showed the lowest figure at 60.1 μΩm. As the pressure increased further, the electrical resistivity slightly increased, but the degree of increase was insignificant.

Electrical resistivity is affected by the presence of pores. As porosity increases, the electrical resistivity increases [35]. Additionally, as the size of porosity increases, the electrical resistivity also increases [36]. In this study, it was observed that the porosity of the isotropic carbon blocks decreased with increasing CIP pressure. CIP-100 exhibited the lowest electrical resistivity; however, its electrical resistivity increased despite the decrease in porosity. In Figure 7, the pores present in CIP-100 are small and not connected to each other. The pores of CIP-150 and CIP-200 were connected to each other by the formation of granules, forming long and large pores. This change in pore size is thought to be responsible for the increase in electrical resistivity.

The average values for properties are shown in Table 1.

### 3.6. Isotropic Ratio

Table 2 presents the CTE of the isotropic carbon blocks fabricated with respect to CIP pressures and from these CTE, isotropic ratios were calculated.

CIP-50 achieved the highest CTE at 5.76 μm/(m·°C) while CIP-100 exhibited the lowest CTE at 5.60 μm/(m·°C). As the applied pressure further increased, the CTE values increased.

The isotropic ratio of the carbon blocks obtained in this study was estimated based on the CTE values. The results confirmed that all carbon blocks obtained proved to be isotropic. In CIP-100 and CIP-200, the average isotropic ratio was closest to 1.00.

## 4. Conclusions

In this study, isotropic carbon blocks were fabricated while varying the applied pressure during the CIP. The effect of CIP pressure on the density, porosity, mechanical and electrical properties, isotropic ratio, and microstructure of the resulting blocks was analyzed. The major findings of the present study are as follows.

As the CIP pressure increased, the bulk density increased while the porosity decreased. Regardless of the CIP pressure, the deviation between density measurements was within 0.02 g/cm^3^.

CIP-100 exhibited the highest flexural strength at 33.3 MPa, along with the highest Shore hardness at 88.2 HsD. As the CIP pressure increased further, both the flexural strength and hardness decreased.

The lowest electrical resistivity was achieved in CIP-100 at 60.1 μΩm, and with a further increase in CIP pressure, the electrical resistivity also tended to increase.

Notably, the observed changes can be explained in the mechanical properties and electrical resistivity resulting with respect to CIP pressures based on the associated changes in the microstructure. As the CIP pressure increased, granules grew larger, and this was accompanied by changes in the arrangement and size of pores around them. In CIP-100, small pores around the granules existed separately without being connected with others. As the CIP pressure increased further, the pores tended to connect with each other and coalesce, serving as crack-like defects.

The isotropic ratio of the carbon blocks obtained in this study was estimated based on the CTE. The results confirm that all carbon blocks obtained proved to be isotropic.

In general, it has been reported that decreasing porosity increases flexural strength [32,33] and decreases electrical resistivity [35]. Based on these reports, we expected that increasing CIP pressure would increase the bulk density of the isotropic carbon block and decrease the porosity, resulting in improved mechanical and electrical properties. However, the results of this study were not as expected. Bulk density increased and porosity decreased with increasing CIP pressure, but the highest flexural strength and lowest electrical resistivity were measured for CIP-100. This is attributed to the formation of large granules during molding at too high a pressure and the formation of long pores around them, which resulted in property degradation. Therefore, to optimize the manufacturing process of graphite blocks, it is necessary to measure the properties of several samples and then obtain and evaluate the deviation. Analysis of properties should be performed by examining the microstructure.

There have been no reports on the fabrication of isotropic carbon blocks as a function of CIP pressure, but we found that 100 MPa was the best pressure condition for the size of the isotropic carbon blocks produced in this study.

The isostatic coke used in this study is prepared by distillation, drying, coking, and calcination using coal tar as a starting material [37,38]. This process produces a wide variety of volatile materials [39]. Most commercial graphite blocks are fabricated using this raw material, and we have adopted the CIP method to improve performance. Recently, various efforts are being made around the world to realize carbon neutrality by utilizing biochar as a raw material [40,41,42], and we are also making efforts to practice in this direction [43].

## Figures and Tables

**Table 1:** Summary of mechanical and electrical properties with respect to CIP pressure.

Properties	Samples			
	CIP-50	CIP-100	CIP-150	CIP-200
Bulk density (g/cm^3^)	1.431	1.477	1.504	1.521
Porosity (%)	27.4	24.9	23.7	22.7
Deviation of density (g/cm^3^)	0.009	0.016	0.019	0.015
Flexural strength (MPa)	23.7	33.3	22.2	19.7
Shore hardness (HsD)	85.7	88.2	87.9	87.2
Electrical resistivity (μΩm)	70.8	60.1	62.9	63.0

**Table 2:** CTE for isotropic carbon blocks with respect to CIP pressure and the isotropic ratio calculated from CTE.

Samples	Axis	CTE (μm/m·°C)		Isotropic Ratio			
		Axis Average	Overall Average	x/z	y/z	y/x	Average
CIP-50	x-axis	5.86	5.76	1.06	1.07	1.01	1.05
	y-axis	5.92					
	z-axis	5.51					
CIP-100	x-axis	5.63	5.60	1.02	1.01	1.00	1.01
	y-axis	5.62					
	z-axis	5.55					
CIP-150	x-axis	5.70	5.68	0.98	0.96	0.98	0.98
	y-axis	5.53					
	z-axis	5.80					
CIP-200	x-axis	5.69	5.68	1.01	1.01	1.00	1.01
	y-axis	5.69					
	z-axis	5.65					

## Data Availability

Data are contained within the article.
